# Hepatitis C, mental health and equity of access to antiviral therapy: a systematic narrative review

**DOI:** 10.1186/1475-9276-12-92

**Published:** 2013-11-18

**Authors:** Julie Hepworth, Tanya Bain, Mieke van Driel

**Affiliations:** 1 School of Public Health and Social Work, Queensland University of Technology, Queensland, Australia; 2 HIV/HCV Education Unit, Discipline of General Practice, School of Medicine, The University of Queensland, Queensland, Australia; 3 Discipline of General Practice, School of Medicine, The University of Queensland, Queensland, Australia

## Abstract

**Introduction:**

Access to hepatitis C (hereafter HCV) antiviral therapy has commonly excluded populations with mental health and substance use disorders because they were considered as having contraindications to treatment, particularly due to the neuropsychiatric effects of interferon that can occur in some patients. In this review we examined access to HCV interferon antiviral therapy by populations with mental health and substance use problems to identify the evidence and reasons for exclusion.

**Methods:**

We searched the following major electronic databases for relevant articles: *PsycINFO, Medline, CINAHL, Scopus, Google Scholar.* The inclusion criteria comprised studies of adults aged 18 years and older, peer-reviewed articles, date range of (2002–2012) to include articles since the introduction of pegylated interferon with ribarvirin, and English language. The exclusion criteria included articles about HCV populations with medical co-morbidities, such as hepatitis B (hereafter HBV) and human immunodeficiency virus (hereafter HIV), because the clinical treatment, pathways and psychosocial morbidity differ from populations with only HCV. We identified 182 articles, and of these 13 met the eligibility criteria. Using an approach of systematic narrative review we identified major themes in the literature.

**Results:**

Three main themes were identified including: (1) pre-treatment and preparation for antiviral therapy, (2) adherence and treatment completion, and (3) clinical outcomes. Each of these themes was critically discussed in terms of access by patients with mental health and substance use co-morbidities demonstrating that current research evidence clearly demonstrates that people with HCV, mental health and substance use co-morbidities have similar clinical outcomes to those without these co-morbidities.

**Conclusions:**

While research evidence is largely supportive of increased access to interferon by people with HCV, mental health and substance use co-morbidities, there is substantial further work required to translate evidence into clinical practice. Further to this, we conclude that a reconsideration of the appropriateness of the tertiary health service model of care for interferon management is required and exploration of the potential for increased HCV care in primary health care settings.

## Introduction

The estimated global prevalence of HCV is approximately 130–170 million with regional variations [1]. Every year around 3–4 million people are infected with HCV and more than 350,000 people die from HCV [2]. HCV is a major cause of morbidity and mortality due to liver disease including inflammation, cirrhosis and hepatocellular carcinoma. The majority of people with HCV are injecting drug users (IDUs) and a significant proportion of those have mental health problems [3,4]. Therefore, HCV involves widespread psychosocial morbidity that can pre-exist diagnosis, and be compounded by or result from the diagnosis of HCV due to it being a life threatening disease. As stated by Hirsch and Wright [5] (2003: 536), *“Hepatitis C is an insidious, slowly progressive killer”.* The standard treatment for HCV is combination therapy with peginterferon and ribavirin (hereafter interferon) with new direct acting antiviral drugs becoming available for patients with specific genotypes [6]. Because interferon is associated with neuropsychiatric adverse effects [7], in earlier clinical practice from around the 1990s, patients with mental health and substance use were often excluded from accessing interferon therapy [8]. Current clinical guidelines demonstrate that such exclusion is no longer necessary except in certain circumstances [9]. However, substantial increased access to interferon by populations with mental health and substance use problems has not been achieved to date. For example, in Australia, only approximately 3,500 people per year commence antiviral therapy, this figure is well below the required 6,000 to stabilize the population from progressing to advanced liver disease [3], and potential adverse health effects post treatment is a deterrent [10]. Therefore, access to HCV treatment and health care services by populations with mental health and substance use problems is complicated by a number of factors and barriers raising a key issue about equity.

As the WHO (2007) maintains, health care systems have a weak health equity orientation, are not pro-poor and do not effectively address the health needs of marginalized groups [11]. In the case of HCV, treating patients with HCV and mental health co-morbidity is already complex, but is further compounded by inadequate health care systems and service delivery. Furthermore, HCV populations can experience stigma and discrimination in both health service use and in the community [12,13]. Common reasons for the exclusion of patients with HCV, mental health and substance use problems to access antiviral therapy include not only the possibility of psychiatric morbidity, but concerns about lack of adherence to the interferon regimen. In a recent study of physicians 48% refused to treat HCV infected patients because of alcohol and drug use, even though drug and alcohol use did not affect sustained virological response, and 46% refused naming mental health reasons as the barrier to treatment [14]. At the primary care level only 39% of general practitioners were highly likely to discuss psychosocial issues with patients and 37% reported difficulty with having a central role in the psychological and medical care of their patients [15].

Therefore, given the complexity of issues involved with HCV, mental health and access to antiviral therapy, we aimed to develop a review summary that would inform current directions for access to antiviral therapy and management of these vulnerable HCV populations. The main objective of this paper was to provide an overview of the main themes in the literature about HCV, mental health and antiviral therapy from which a critical discussion could be developed rather than an exhaustive systematic review. The main research question was: How do mental health and substance use co-morbidities affect access to interferon therapy?

## Method

### Search strategy

We searched the following major electronic databases for relevant articles: *PsycINFO, Medline, CINAHL, Scopus, Google Scholar.* The inclusion criteria comprised adults aged 18 years and older, peer-reviewed articles, date range of (2002–2012) to include articles since the introduction of pegylated interferon with ribarvirin, and English language. The exclusion criteria included articles about HCV populations with medical co-morbidities, such as HBV and HIV, because the clinical treatment, pathways and psychosocial morbidity differ from populations with only HCV. The search terms were “hepatitis C” AND “psychosocial” AND “treatment” (and multiple variations of search terms as listed in Appendix 1) and to appear anywhere in the title or abstract. To extend the search for specific relevant articles we also used snowball sampling to identify key articles from the reference lists of articles we had already obtained. Articles were eligible if they referred to populations who were being considered for interferon therapy and who had a mental health diagnosis or both a mental health diagnosis and substance/alcohol use problems in the title and/or abstract. The focus of the review was on access to interferon antiviral therapy by these populations. The following types of articles were included: original research, literature reviews, clinical reviews, expert opinion and a consensus statement.

We organized the data according to its relationship to access to antiviral therapy categorizing it into three main areas: (1) preparation for antiviral therapy, (2) during treatment and adherence to treatment, and (3) clinical outcomes. These areas represented the major foci that inform clinical practice around access to HCV antiviral therapy. This categorization provided a framework with which to critically discuss the literature and identify gaps in research, health services and clinical practice. Narrative review frameworks informed the review [16,17]. A particular strength of meta-narrative review is that it enables researchers to demonstrate how a phenomenon has unfolded over time and the sorts of questions that have been asked at particular points in time [16]. This approach to a review was useful for the area of HCV, mental health and access to treatment because it enabled us to identify the complexities and barriers related to the exclusion of people with mental health and/or substance use problems from accessing antiviral therapy. By drawing on work by Greenhalgh et al. [17] we used the six phases in narrative review to guide a systematic approach to the selection and analysis of key literature in the field, and summarise these phases in Table 1.

**Table 1:** Summary of the phases in meta-narrative review (adapted from Greenhalgh et al. 2005)

	**Greenhalgh et al. **[17]	**Adapted**
1	Planning phase: assemble multidisciplinary team, establish regular meetings.	→ Established multidisciplinary team.
		→ Held regular team meetings.
2	Search phase: initial search led by intuition, search for seminal papers, search for empirical papers.	→ Systematic search of major electronic databases.
		→ Searched for seminal papers.
3	Mapping phase: identify key elements of research traditions, main findings.	→ Identified key approaches to the problem of access to HCV antiviral therapy.
		→ Identified inconsistencies across approaches.
4	Appraisal phase: evaluation of each paper for relevance to the review question, extract key results, group comparable results;	→ Evaluated each paper for relevance to the research question, extracted key results and categorized comparable areas.
5	Synthesis phase: identify all key dimensions of the problem, give a narrative account of each contribution, treat conflicting findings as higher order data and explain;	→ Described each area with reference to included articles.
		→ Critically discussed the areas and inconsistencies across the articles.
6	Recommendations phase: summarise overall messages from the research literature, distil and discuss recommendations for practice, policy and further research.	→ Summarised and distilled key messages from the literature.
		→ Made recommendations for developing clinical guidelines and health service policy.

The multidisciplinary research team comprised a health psychologist, an academic general practitioner, and a specialist HCV researcher and policy analyst. The team held regular meetings to develop and refine the research question, the search terms, discuss emerging findings and analyse key articles. The articles were read several times by the authors, repeatedly checked for their relevance to the research question and search terms. Based on these analyses it was possible to identify key narratives and build a consensus among the research team about the areas of major interest in the literature (see Table 2 below). Subsequently, the research team critically discussed the findings and formulated recommendations.

**Table 2:** Key narratives in research on HCV, mental health and antiviral therapy

**Research focus**	**Author**	**Article type**	**Primary purpose**	**Outcomes/conclusion**
Preparation for HCV antiviral	Bonner et al. [18]	Review of clinical experience	This paper sought to highlight critical pre-treatment strategies and provide tangible resources for HCV clinicians to facilitate preparation and successful treatment of these patients.	HCV clinicians (gastroenterologists/hepatologists) are in a unique position to prepare patients with co-existing MH and/or SA issues for antiviral therapy. Safely treat these populations with multidisciplinary care. Specialist, hospital clinicfocus.
	Hong et al. [19]	Clinical case study	Case presentation of a 50 year-old man with HCV and an extensive history involving alcoholism,depression, and suicidiality who participated in a psycho-education group to help prepare him for treatment with pegylated alpha/ribavirin interferon therapy.	Psycho-education groups show promise to prepare patients for intensive medical treatment. The challenge is to help patients overcome barriers to treatment, particularly psycho-social problems, because available treatments are increasingly effective.
	Rifai et al. [20]	Literature Review (1972-2009)	Review summary of the psychiatric implications of HCV infection and strategies for the management of interferon alfa-induced neuropsychiatric adverse effects.	Interferon can be safely administered to patients with psychiatric disorders provided there is comprehensive pre-treatment assessment, a risk-benefit analysis, and intensive ongoing medical and psychiatric follow-up.
	Sylvestre & Zweben [30]	Descriptive report	Report of a peer-based HCV model to address barriers to treatment intervention.	Peer-based model was successful at engaging, educating, and treating drug users and can facilitate their successful screening and treatment
	Knott et al. [21]	Evaluation study	Evaluation of the effect of integrating psychiatric and medical care on evaluation for and initiation of antiviral treatment.	An integrated MH and medical approach was associated with rates of antiviral therapy recommendation and initiation similar to patients without risk for psychiatric or substance use problems.
Adherence/completion antiviral therapy	Norman et al. [22]	Research article	Description of an evaluation of a peer-based integrated model of care.	A high level of patient acceptability by patients using the service.
	Dollarhide et al. [23]	Retrospective chart review	To evaluate the impact of common psychiatric disorders on treatment completion of antiviral therapy prescribed to a series of hepatitis C (virus) positive US veterans.	Prior psychiatric or substance use history did not predict completion of recommended IFN/ribavirin treatment. Findings. suggest a larger pool of veterans with psychiatric or substance use disorders may be considered for antiviral therapy when provided with multidisciplinary support.
Clinical outcomes	Schaefer et al. [24]	Meeting report/EU Consensus statement	Summary of current knowledge of HCV infection, antiviral treatment and mental health.	The experience of the last 10 years has clearly shown that patients with psychiatric co-morbidity should not necessarily be excluded from IFN-a-based antiviral therapy.
	Freedman & Nathanson [25]	Literature review (2003-2007)	Review of evidence-based best clinical practice of HCV with IFN-based therapy in patients with severe mental illness (SMI) and substance use disorders (SUDs).	clinical outcomes comparable with those without these comorbidities.
	Schaefer et al. [26]	Prospective study	Investigated and compared the results of treating the chronic hepatitis C (HCV) infection of different groups of psychiatric-risk patients and controls with pegylated interferon alpha plusribavirin.	Psychiatric diseases and/or drug addiction did not negatively influence psychiatric tolerability of and antiviral response rate to HCV treatment with pegylated interferon alpha plus ribavirin.
	Mistler [27]	Clinical case report	Report on three patients with hepatitis C infection, severe mental illness, and substance use disorders.	Patients were successfully treated for hepatitis C (cleared the virus) with carefully monitoring and psychiatric oversight.
	Sylvestre et al. [28]	Conference report	Summarises current management issues.	Selected substance users can be candidates for HCV treatment even in the setting of psychiatric disease and relapse to drug use.
	Loftis & Hauser [8]	Review	Examines co-management models of care for HCV patients with psychiatric and substance use.	Many patients with comorbid use diagnoses can be treated safely and effectivelypsychiatric and substance with co-management strategies.

## Results

**Figure 1:**
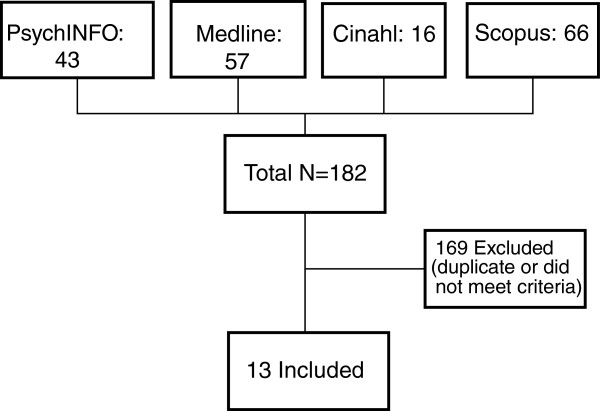
Literature searching strategy.

The initial search retrieved 182 articles, 169 were excluded because they did not meet the inclusion criteria or duplicated articles, resulting in a total of 13 included articles (see Figure 1). These included literature reviews (n = 3), clinical reviews/reports (n = 3), description of a clinical model (n = 1), evaluation (n = 1), retrospective chart review (n = 1), prospective study (n = 1), qualitative research (n = 1), and consensus statement/conference report (n = 2). All articles referred to populations with mental health and substance use problems.

Three key areas were identified:

(1) Pre-treatment strategies/preparation for HCV antiviral therapy.

Preparation for antiviral therapy included psycho-education, comprehensive psychiatric/psychological and medical screening and assessment, and the establishment of collaborative care [18,19]. The periods of time in preparation for HCV therapy varied considerably from several weeks to several months, and involved the delivery of programs that had been specifically designed to prepare patients for HCV antiviral therapy and/or the referral of patients between psychiatric and medical specialists for assessment [18,20,21]. In the latter case, the type of care that was provided was referred to as multidisciplinary [18] but primarily involved a medical specialist located in a hospital who sought mental health and substance abuse services in the community as required rather than the involvement of a multidisciplinary team from the outset of management. This is important because integrated multidisciplinary team care is argued to result in more effective care, greater patient satisfaction and reduces the risk of a dislocation of communication and care coordination across hospital and community based services.

(2) Adherence/completion of antiviral treatment.

Adherence to HCV medical regimens and the completion of treatment by patients with mental health and/or substance use problems have been concerns in the HCV literature for over two decades. The articles (n = 2) that focused on adherence/completion of antiviral therapy demonstrated that mental health and/or substance use did not affect completion rates [22,23]. Additionally, this research prioritized engagement with patients and easier access to health services by HCV populations through community-based settings such as drug and alcohol clinics resulting in increased adherence and treatment completion.

(3) Clinical outcomes and health service delivery.

In terms of clinical outcomes, 6 articles make clear conclusions that mental health and substance use do not have a deleterious effect and outcomes were comparable to populations without these co-morbidities [8,24-28]. The major setting for the management of HCV, mental health and antiviral therapy was a hospital specialist outpatient liver clinic in 8 articles [8,21,23-28], in 2 articles the model of care was community-based [22,29], and in 1 article [8] various settings were included. In the case presentation involving psycho-education [19] models of care was not relevant, and the consensus statement on HCV, mental health and antiviral therapy did not address models of health service delivery [24]. Multidisciplinary teams were reported in all articles and their composition ranged from the inclusion of a psychiatrist in the liver clinic for routine psychiatric assessment pre and during treatment through to broader teams that included any or all of the following: liver specialist, an addiction specialist, primary care physician/general medical practitioner, nurse, and HCV peer worker. The broader multidisciplinary team composition was evident in 9 [8,18-23,27,30] of the 13 articles.

## Discussion

This review has identified emerging research evidence that overall populations with mental health and substance/alcohol use problems can and should have similar access to interferon as populations who do not have these comorbidities. In contrast to some clinical practices that have excluded populations with mental health and/or substance use problems from accessing interferon therapy, this review provides clear evidence based on original research, clinical observations and clinical reviews that these populations can be safely treated in a multidisciplinary care context [18-20,24,27,28,30], that prior psychiatric or substance use history does not predict completion of IFN/ribavirin treatment [23], and that clinical outcomes are comparable with those without these co-morbidities [25]. Of particular note is the recent European Expert Consensus statement [24] that maintains patients with pre-existing disorders can be treated with interferon based regimens and do not have an increased poor compliance, lower SVR, more severe depression during treatment or treatment failure. This finding is supported by some clinical guidelines for the management of HCV patients [9].

This evidence has fundamental implications for equity in relation to patient access to health care, for health professionals, for the implementation of clinical guidelines for HCV management, and for health system change. Having clear evidence to support such widespread increased access to interferon, however, is not always translated into clinical practice. A number of reasons may account for the lack of evidence-based practice, including clinicians’ entrenched beliefs about populations with mental health/substance use problems being barriers to effective treatment, discrimination, economic considerations, lack of appropriate health service delivery and poor patient engagement.

Unlike the requirement that interferon commencement is conditional upon demonstrable improvement in patients’ mental health and/or abstinence from alcohol/substance use, in the above studies interferon was commenced within a period ranging from several weeks or months. This demonstrates a shortening of the length of time for treatment to commence from what was typically 6 months. During this period comprehensive pre-HCV therapy strategies were employed involving one or more of the following: (1) mental health and substance use screening and assessment, (2) HCV/IFN information, education and support, and (3) establishment of multidisciplinary care [18-20,22-25,27,28,30]. Comprehensive regular monitoring was continued during and post interferon therapy. In cases where there was uncontrolled major depression adequate symptom remission was necessary prior to commencing interferon therapy [23]. These studies demonstrate a different approach to patient access to interferon whereby on participation in or completion of a clearly defined pre-therapy preparatory program patients progress to commence therapy. Therefore, there is considerable variation across different global regions regarding the clinical requirement for some degree of remediation or rehabilitation from the patient in order to receive treatment. This suggests a need for changing some clinicians’ values and perceptions of these population groups to enable both an increase in the expansion of access to interferon treatment and earlier commencement of treatment.

The most common health care setting in which interferon was managed in the above studies was a specialist clinic in a major tertiary hospital. All studies refer to a multidisciplinary and/or integrated model of care for the management of interferon with patients with mental health and substance/alcohol use problems largely comprising, however, only a gastroenterologist or HCV/liver specialist and a psychiatrist [18,19,23,24,27]. In the study by Dollarhide et al. [23] the multidisciplinary team was expanded to include an addiction specialist. The study by Rifai et al. [20] was the only one to report inclusion of a primary care physician.

Primary care generally provides low threshold, highly accessible and multidisciplinary comprehensive care with a focus on delivering high quality chronic disease management. It could therefore be an ideal environment to ensure management of chronic care services to vulnerable population groups such as HCV patients. However, the only other model of HCV interferon management identified was community based health care located in drug and alcohol clinics. In the study by Norman et al. [22] they demonstrate that it is feasible to provide a quality HCV treatment service that had a high acceptability to substance using clients. The integrated multidisciplinary team included primary care medical practitioners, a visiting specialist liver physician, peer worker, nurse, pharmacist and allied health as required. Similarly, Sylvestre et al. [28] maintain that interferon treatment can be successfully integrated into health care settings that provide care for populations with mental health and substance use problems such as methadone clinics, prisons and community-based ‘walk-in’ clinics. Arora et al. [29] have combined integrated multidisciplinary care with videoconferencing to establish and increase access to HCV care in marginalized communities with significant improvements in providers’ experiences of care delivery. Finally, Sylvestre and Zweben [30] report positive outcomes from a community-based program based on collaboration between medical providers and peer educators whereby the meaningful engagement of IDUs enabled screening and HCV treatment resulting in further positive effects on adherence. Currently, there appears to be some emerging randomized controlled trials of integrated care such as those by Evon et al. [31] and Groessl et al. [32] but these are restricted to major medical centres, and there is little focus on the potential of integrated multidisciplinary care of HCV management in primary health care settings. The emerging studies of community-based models of health care for interferon management were restricted to injecting drug user settings and no studies involving general practice were identified. Indeed, there is a paucity of research on the potential for interferon to be managed in general practice or other primary care settings. General practitioners, however, are reportedly not confident to initiate therapy, but do have an interest in education about HCV antiviral therapy [33] demonstrating the need for more research investment into their education and service development needs.

The dominance of hospital managed HCV antiviral treatment clearly serves as a major barrier to the populations most affected by HCV; people with mental health and substance use co-morbidities. The research evidence suggests that for these populations it is imperative to establish their engagement through community-based clinics run by broad-based multidisciplinary teams. Therefore, we identified in this review an inverse relationship between the type of health service model best suited to HCV, mental health and substance use populations and what is actually available as the dominant model of care. The hospital specialist model was designed around the needs and convenience of medical specialists rather than those of the population groups and patient-centric care. We acknowledge that new medicines such as those on their way for HCV require specialist oversight when introduced, but for the majority of HCV populations this is not the case. The prior example of the transition of the management of insulin initiation away from hospitals and towards community-based clinics serves as an analogy for what we observe is unfolding in the area of HCV. In terms of models of service delivery, the Beacon practice model [34], could be a framework for innovative HCV health service research to address increased access. As part of future models of HCV management it is also necessary to consider the introduction of interferon free HCV antiviral treatment regimens. However, given the current speculation about the timing of the availability of new HCV medications interferon may well continue to be the major treatment for some time yet, although the prescribing practice will be of significant interest in terms of access and equity.

This review drew on guiding principles of narrative review and in so doing involved searching the international literature. One of the limitations of the review was that the term ‘psychosocial’ was particularly problematic for a review using systematic principles because it has several different meanings in the HCV literature. Three particular types of use of the term ‘psychosocial’ were identified; first, it is used as an overarching term for one or more psychiatric/psychological diagnoses, and specifically, anxiety, depression, schizophrenia, psychosis, and co-morbidity of diagnoses. Second, in many articles mental health diagnoses were referred to as ‘psychiatric’ whereas ‘psychosocial’ referred to socio-economic status, unemployment, poor social support and even “chaotic living”. Third, in some articles mental health diagnoses were included within the term psychosocial *together with* socio-economic factors. Further to this, our search resulted in only 13 eligible articles and 2 of these were conference reports. Consequently, the extent to which the review identified the current evidence on HCV, mental health and access to interferon remains relatively opaque.

### Recommendations

The promising early results emerging from community-based multidisciplinary care indicate that it would be timely to explore multidisciplinary, integrated interferon management in primary care. The potential benefits of this model of care are threefold; First, it would create a shift away from hospital-based health service delivery toward primary health care as the driver of service provision together with specialist input consistent with international health policy reforms [35,36]. Second, the reorientation of health service delivery is likely to result in considerable cost savings. Third, the expansion of community-based interferon management would facilitate increased equity and access to health services for marginalized populations.

So what is required to create such a change in health service delivery that could increase equity of access to interferon? First, general practitioners need to be motivated, educated and supported to lead comprehensive HCV treatment including interferon management in close collaboration with liver and mental health specialists. Second, there needs to be a systematic exploration of models of multidisciplinary, integrated care in the community-based setting to determine what constitutes quality, safety and the most cost effective model to manage the multiple psychosocial and medical needs of HCV patients. Third, patient engagement is crucial to any successful expansion of access to interferon through community-based services. This involves regular, extensive and well-informed interaction with HCV populations that builds trust between clinic staff and patients through which patients are prepared for interferon therapy, its potential side effects, adherence, and also the possibility of treatment failure.

## Conclusion

This review of access to HCV antiviral therapy by populations with mental health and substance use co-morbidities illustrates that there are very few clinical contraindications to treatment commencement, and, therefore, an increased number of people with HCV could be receiving treatment. Current evidence-based guidelines recommend inclusive access to treatment. However, while there is evidence of a changing perspective in this area, several barriers to increasing access to interferon continue to exist. It is concluded that a concerted effort to research and trial health service development in primary health care settings for the management of HCV antiviral therapy may be beneficial for increasing patient access, treatment completion and contribute to a more equitable health system designed around the needs of patient-centred care.

## Appendix 1

### Barrier terms

barrier* OR deffer* OR coexisiting OR co-exisiting OR vulnerabl* OR obstacle* OR psycho* OR withhold OR “with hold” OR withheld OR “with held” OR decline* OR “non treatment” OR “not treat*” OR contraindicat* OR uptake* OR “up take” OR access* OR untreated OR “non attendance” OR “non referral*” OR marginal* OR “social implication*” OR homeless OR “home less” OR stigma OR refus* OR problem* OR “social implication*” OR stigma* OR equity OR socio* OR co-occuring OR coexisiting OR complex* OR challeng* OR socioeconomic OR finance OR “mental health” OR anxi* OR depress*.

### Treatment terms

(treat* OR antiviral OR therap* OR interferon OR program* OR educat*).

### Hepatitis terms & subject headings

HCV OR “hepatitis C” OR “hep C

(MH “Hepatitis C”) OR (MH “Hepatitis C, Chronic”).

## Competing interests

The authors declare that they have no competing interests.

## Authors’ contributions

All authors participated in the conception and design of the study, analysis of the literature, contributed to drafts of the manuscript and read and approved the final manuscript.
